# Supplementary material for: Would Surgeons Like to Be Submitted to Anal Fistulotomy? An International Web-Based Survey
Source: J Clin Med. 2023 Jan 20;12(3):825. doi: 10.3390/jcm12030825 (PMC9918049; doi:10.3390/jcm12030825)
Supplement: Supplementary file 1 [file jcm-12-00825-s001.zip › jcm-2167901-supplementary.pdf]

SUPPLEMENTARY MATERIALS

Table S1. The online questionnaire was composed by four sections on participants' demographics, professional experience in fistula surgery and opinions about psychological and surgical aspects either in low or high anal fistula.

**SECTION I (Respondents' demographics)**					
**Gender**:	Male	Female			
**In which country do you practice?**					
**Type of hospital**:	Academic	Non-academic teaching	Non-teaching		
**Training level**:	Consultant	Resident	Fellow		
**Age (years)**:	<30	30-39	40-49	50-59	60 or older

**SECTION II (Professional experience in fistula surgery)**							
**Years of professional experience in anal fistula management:**	0-5	6-10	11-20	>20			
**Personal experience in anal fistula management during the last year (number of cases):**	None	1-10	11-20	21-30	31-40	41-50	>50

**SECTION III (Try to imagine to be a patient with a low anal fistula)**					
**Do you feel that the presence of the anal fistula is able to negatively affect your quality of life, limiting/reducing/modifying your daily activities?**	Very much	Much	Not too much	Just a little	Not at all
**Are you worried/anxious because of your anal fistula?**	Very much	Much	Not too much	Just a little	Not at all
**Taking into account that the fistulotomy for you would give percentages of healing of 85-98%, and, then, percentages of failure of 2-15%, do you consider this perspective acceptable?**	Yes, absolutely	Enough acceptable	Even if high rates of success, the percentages of possible failure are still considerable	Not too much	Not at all
**Would you be worried about the possibility to be submitted to more than one surgery to treat your anal fistula?**	Very much	Much	Not too much	Just a little	Not at all
**Taking into account that the fistulotomy for you could give some impairment of continence (occurring in about 6-28% of cases, and consisting mainly in gas incontinence or post-defecation staining in the underwear, through inadvertent passage of flatus, and, sometimes, also leakage of liquid or solid stools), do you consider this perspective acceptable?**	Yes, absolutely	Enough acceptable	Even if high rates of success, the percentages of possible failure are still considerable	Not too much	Not at all
**Finally, taking into account all the data concerning fistulotomy to treat an anal fistula like that you have, would you agree to be submitted to the fistulotomy?**	Yes	No			

**SECTION IV (Try to imagine to be a patient with a high anal fistula)**					
**Do you feel that the presence of the anal fistula is able to negatively affect your quality of life, limiting/reducing/modifying your daily activities?**	Very much	Much	Not too much	Just a little	Not at all
**Are you worried/anxious because of your anal fistula?**	Very much	Much	Not too much	Just a little	Not at all
**Taking into account that the fistulotomy for you would give percentages of healing of 85-98%, and, then, percentages of failure of 2-15%, do you consider this perspective acceptable?**	Yes, absolutely	Enough acceptable	Even if high rates of success, the percentages of possible failure are still considerable	Not too much	Not at all
**Would you be worried about the possibility to be submitted to more than one surgery to treat your anal fistula?**	Very much	Much	Not too much	Just a little	Not at all
**Taking into account that the fistulotomy for you could give some impairment of continence (occurring in about 17.5-40% of cases, and consisting mainly in gas incontinence or post-defecation staining in the underwear, through inadvertent passage of flatus, and, sometimes, also leakage of liquid or solid stools), do you consider this perspective acceptable?**	Yes, absolutely	Enough acceptable	Even if high rates of success, the percentages of possible failure are still considerable	Not too much	Not at all
**Finally, taking into account all the data concerning fistulotomy to treat an anal fistula like that you have, would you agree to be submitted to the fistulotomy?**	Yes	No			

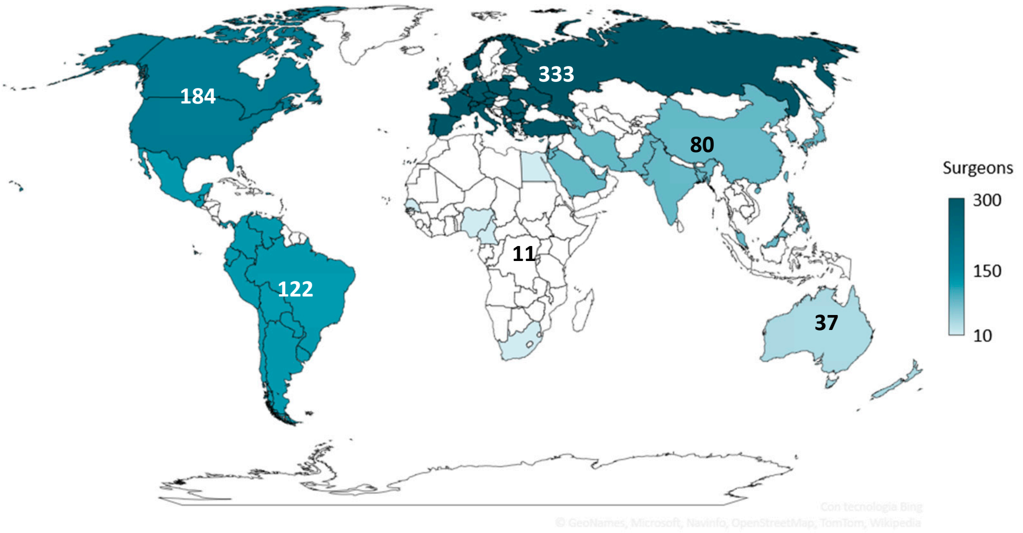

Figure S1. Geographical distribution of 767 surgeons who completed the survey on anal fistulotomy.

Table S2. Geographical distribution per continents of 767 surgeons who would accept fistulotomy in low and high anal fistula separately, compared to surgeons who would do that in the rest of the World (chi-squared test). The *p*-value in bold was statistically significant.

	LOW FISTULA			HIGH FISTULA		
**Geographical distribution (*n*° respondents)**	**Would accept fistulotomy (%)**	**Would accept fistulotomy in the rest of the WORLD (%) ***	**p-value**	**Would accept fistulotomy (%)**	**Would accept fistulotomy in the rest of the WORLD (%) ***	**p-value**
Europe (333)	278 (83.5)	393 (90.6)	**0.003**	120 (36.0)	170 (39.2)	0.375
Africa (11)	9 (81.8)	662 (87.6)	0.567	4 (36.4)	286 (37.8)	0.921
Asia (80)	67 (83.8)	604 (87.9)	0.286	36 (45.0)	254 (37.0)	0.161
Oceania (37)	30 (81.1)	641 (87.8)	0.228	4 (10.8)	286 (39.2)	**0.001**
North America (184)	174 (94.6)	497 (85.2)	**0.001**	69 (37.5)	221 (37.9)	0.921
Middle-South America (122)	113 (92.6)	558 (86.5)	0.061	57 (46.7)	233 (36.1)	**0.027**

*In this column, statistical analyses were performed considering data of the overall sample less the answers of respondents from the single continent indicated in the corresponding row.

Table S3. Geographical distribution per countries of surgeons who would accept fistulotomy in low and high anal fistula separately, compared to surgeons who would do that in the rest of the World (chi-squared test). The *p*-value in bold was statistically significant.

	**LOW FISTULA**			**HIGH FISTULA**		
	**Would accept fistulotomy (%)**	**Would accept fistulotomy in the rest of the WORLD (%) ***	**p-value**	**Would accept fistulotomy (%)**	**Would accept fistulotomy in the rest of the WORLD (%) ***	**p-value**
Albania	1 (100)	670 (87.5)	0.705	1 (100)	289 (37.7)	0.199
Argentina	14 (100)	657 (87.3)	0.153	5 (35.7)	285 (37.8)	0.870
Aruba	1 (100)	670 (87.5)	0.705	0 (0)	290 (37.9)	0.435
Australia	24 (77.4)	647 (87.9)	0.084	**2 (6.5)**	**288 (39.1)**	**<0.001**
Austria	1 (100)	670 (87.5)	0.705	0 (0)	290 (37.9)	0.435
Azerbaijan	1 (100)	670 (87.5)	0.705	1 (100)	289 (37.7)	0.199
Bangladesh	1 (100)	670 (87.5)	0.705	1 (100)	289 (37.7)	0.199
Belgium	4 (80.0)	667 (87.5)	0.612	0 (0)	290 (38.1)	0.080
Bolivia	2 (100)	669 (87.5)	0.592	1 (50.0)	289 (37.8)	0.722
Brazil	18 (85.7)	653 (87.5)	0.804	11 (52.4)	279 (37.4)	0.163
Bulgaria	4 (100)	667 (87.4)	0.448	2 (50.0)	288 (37.7)	0.614
Cameroon	1 (100)	670 (87.5)	0.705	0 (0)	290 (37.9)	0.435
Canada	16 (94.1)	655 (87.3)	0.403	4 (23.5)	286 (38.1)	0.219
Chile	4 (100)	667 (87.4)	0.448	1 (25.0)	289 (37.9)	0.596
China	2 (66.7)	669 (87.6)	0.275	1 (33.3)	289 (37.8)	0.873
Colombia	6 (100)	665 (87.4)	0.352	4 (66.7)	286 (37.6)	0.143
Cyprus	1 (100)	670 (87.5)	0.705	0 (0)	290 (37.9)	0.435
Czech Republic	1 (50.0)	670 (87.6)	0.109	1 (50.0)	289 (37.8)	0.722
Denmark	2 (100)	669 (87.5)	0.592	0 (0)	290 (37.9)	0.270
Ecuador	3 (100)	668 (87.4)	0.512	2 (66.7)	288 (37.7)	0.302
Egypt	7 (100)	664 (87.4)	0.315	4 (57.1)	286 (37.6)	0.289
UK	21 (87.5)	650 (87.5)	0.998	8 (33.3)	282 (38.0)	0.646
Finland	**0 (0)**	**671 (87.7)**	**<0.001**	0 (0)	290 (37.9)	0.270
France	13 (86.7)	658 (87.5)	0.923	4 (26.7)	286 (38.0)	0.369
Germany	12 (92.3)	659 (87.4)	0.596	3 (23.1)	287 (38.1)	0.269
Greece	8 (100)	663 (87.4)	0.282	4 (50.0)	286 (37.7)	0.475
Guatemala	1(100)	670 (87.5)	0.705	0 (0)	290 (37.9)	0.435
Hong Kong	3 (100)	668 (87.4)	0.512	1 (33.3)	289 (37.8)	0.873
India	7 (100)	664 (87.4)	0.315	5 (71.4)	285 (37.5)	0.065
Iran	1 (50.0)	670 (87.6)	0.109	0 (0)	290 (37.9)	0.270
Ireland	4 (100)	667 (87.4)	0.448	3 (75.0)	287 (37.6)	0.124
Israel	3 (75.0)	668 (87.5)	0.449	2 (50.0)	288 (37.7)	0.614
Italy	64 (90.1)	607 (87.2)	0.478	32 (45.1)	258 (37.1)	0.185
Japan	6 (75.0)	665 (87.6)	0.283	4 (50.0)	286 (37.7)	0.475
Jordan	3 (100)	668 (87.4)	0.512	2 (66.7)	288 (37.7)	0.302
Kuwait	2 (100)	669 (87.5)	0.592	1 (50.0)	289 (37.8)	0.722
Lebanon	2 (100)	669 (87.5)	0.592	1 (50.0)	289 (37.8)	0.722
Lithuania	1 (100)	670 (87.5)	0.705	0 (0)	290 (37.9)	0.435

Malaysia	**0 (0)**	**671 (87.6)**	**0.008**	0 (0)	290 (37.9)	0.435
Mexico	55 (93.2)	616 (87.0)	0.166	28 (47.5)	262 (37.0)	0.112
Netherlands	11 (91.7)	660 (87.4)	0.659	2 (16.7)	288 (38.1)	0.128
New Zealand	6 (100)	665 (87.4)	0.352	2 (33.3)	288 (37.8)	0.820
Nigeria	1 (100)	670 (87.5)	0.705	0 (0)	290 (37.9)	0.435
Norway	1 (50.0)	670 (87.6)	0.109	0 (0)	290 (37.9)	0.270
Pakistan	4 (80.0)	667 (87.5)	0.612	**4 (80.0)**	**286 (37.5)**	**0.051**
Panama	2 (100)	669 (87.5)	0.592	0 (0)	290 (37.9)	0.270
Paraguay	2 (100)	669 (87.5)	0.592	2 (100)	288 (37.6)	0.069
Peru	1 (100)	670 (87.5)	0.705	1 (100)	289 (37.7)	0.199
Philippines	14 (93.3)	657 (87.4)	0.489	**2 (13.3)**	**288 (38.3)**	**0.048**
Poland	2 (100)	669 (87.5)	0.592	1 (50.0)	289 (37.8)	0.722
Portugal	6 (100)	665 (87.4)	0.352	1 (16.7)	289 (38.0)	0.284
Puerto Rico	1 (50.0)	670 (87.6)	0.109	1 (50.0)	289 (37.8)	0.722
Qatar	1 (100)	670 (87.5)	0.705	0 (0)	290 (37.9)	0.435
Romania	7 (87.5)	664 (87.5)	0.999	**6 (75.0)**	**284 (37.4)**	**0.029**
Russia	**47 (72.3)**	**624 (88.9)**	**<0.001**	**34 (52.3)**	**256 (36.5)**	**0.012**
Saudi Arabia	5 (100)	666 (87.4)	0.396	2 (40.0)	288 (37.8)	0.919
Senegal	**0 (0)**	**671 (87.6)**	**0.008**	0 (0)	290 (37.9)	0.435
Serbia	4 (100)	667 (87.4)	0.448	0 (0)	290 (38.0)	0.118
Singapore	4 (80.0)	667 (87.5)	0.612	3 (60.0)	287 (37.7)	0.305
Slovenia	1 (100)	670 (87.5)	0.705	0 (0)	290 (37.9)	0.435
South Africa	**0 (0)**	**671 (87.6)**	**0.008**	0 (0)	290 (37.9)	0.435
South Korea	1 (50.0)	670 (87.6)	0.109	1 (50.0)	289 (37.8)	0.722
Spain	34 (87.2)	637 (87.5)	0.953	**8 (20.5)**	**282 (38.7)**	**0.022**
Switzerland	**9 (60.0)**	**662 (88.0)**	**0.001**	3 (20.0)	287 (38.2)	0.151
Taiwan	1 (100)	670 (87.5)	0.705	1 (100)	289 (37.7)	0.199
Trinidad e Tobago	1 (100)	670 (87.5)	0.705	1 (100)	289 (37.7)	0.199
Turkey	20 (80.0)	651 (87.7)	0.250	7 (28.0)	283 (38.1)	0.304
UAE	**5 (62.5)**	**666 (87.7)**	**0.032**	4 (50.0)	286 (37.7)	0.475
Ukraine	**0 (0)**	**671 (87.6)**	**0.008**	0 (0)	290 (37.9)	0.435
Uruguay	1 (100)	670 (87.5)	0.705	0 (0)	290 (37.9)	0.435
USA	**158 (94.6)**	**513 (85.5)**	**0.002**	65 (38.9)	225 (37.5)	0.737
Venezuela	1 (50.0)	670 (87.6)	0.109	0 (0)	290 (37.9)	0.270

*In this column, statistical analyses were performed considering data of the overall sample less the answers of respondents from the single country indicated in the corresponding row.

UK= United Kingdom; UAE= United Arab Emirates; USA= United States of America.
